# Venoarterial extracorporeal membrane oxygenation for peripartum cardiomyopathy complicated by severe preeclampsia: a case report with 5-year follow-up

**DOI:** 10.3389/fmed.2026.1952278

**Published:** 2026-09-04

**Authors:** Haiqing Wang, Mulan Liu, Yixuan Cheng, Xinyu Fang, Zhurui Chen, Jianhao Sun, Dan Lu

**Affiliations:** 1 Northern Jiangsu People's Hospital Affiliated to Yangzhou University, Yangzhou, China; 2 Institute of Translational Medicine, Medical College, Yangzhou University, Yangzhou, China; 3 The Taixing People's Hospital, Taixing, China; 4 The Yangzhou School of Clinical Medicine of Dalian Medical University, Yangzhou, China; 5 The Yangzhou Clinical Medical College of Xuzhou Medical University, Yangzhou, China

**Keywords:** acute heart failure, long-term follow-up, peripartum cardiomyopathy, severe pre-eclampsia, venoarterial extracorporeal membrane oxygenation

## Abstract

**Background:**

Peripartum cardiomyopathy (PPCM) coexisting with severe preeclampsia is uncommon and diagnostically challenging because dyspnea, pulmonary edema, and cardiac dysfunction may be attributed to either condition. Long-term outcomes after rescue mechanical support remain poorly documented.

**Case presentation:**

A 28-year-old primigravida at 37 + 6 weeks presented with 2 days of worsening orthopnea, dyspnea, and cough. At 15 + 3 weeks, electrocardiography had shown complete left bundle branch block, although left ventricular ejection fraction (LVEF) was 61%. On admission, blood pressure was 159/120 mmHg, heart rate 157 beats/min, urine protein was positive, N-terminal pro-B-type natriuretic peptide (NT-proBNP) was 5,660 pg/ml, and echocardiography showed an LVEF of 26%. Severe preeclampsia and acute heart failure due to PPCM were diagnosed. Labetalol, magnesium sulfate, and furosemide were administered, followed by emergency cesarean delivery of a 2,880-g male infant (Apgar scores, 8 and 10). Persistent postoperative hemodynamic instability despite mechanical ventilation and pharmacotherapy prompted multidisciplinary initiation of venoarterial extracorporeal membrane oxygenation (VA-ECMO). She was successfully decannulated on day 4 and discharged on day 21 with an LVEF of 44%. NT-proBNP normalized by 6 months, LVEF exceeded 50% by 9 months, and at 5 years LVEF was 59% and NT-proBNP was 20.9 pg/ml.

**Conclusion:**

This case provides rare 5-year documentation of sustained myocardial recovery after VA-ECMO in PPCM with severe preeclampsia. In peripartum patients with severe hypertension and respiratory symptoms, an LVEF below 45% should prompt evaluation for PPCM; timely echocardiography, multidisciplinary care, and early VA-ECMO may bridge refractory hemodynamic instability to recovery, followed by prolonged cardiac surveillance.

## Introduction

Peripartum cardiomyopathy (PPCM) is a potentially life-threatening idiopathic cardiomyopathy that develops toward the end of pregnancy or within the first 5 months postpartum in women without pre-existing structural heart disease or another identifiable cause of heart failure. PPCM is a diagnosis of exclusion characterized by left ventricular systolic dysfunction and a left ventricular ejection fraction (LVEF) < 45% (1, 2). Preeclampsia with severe features is a hypertensive disorder of pregnancy associated with substantial maternal and fetal morbidity and mortality (3). Although PPCM coexisting with severe preeclampsia is uncommon, differentiating between these conditions can be challenging because both may manifest as dyspnea, pulmonary edema, and cardiac dysfunction. This clinical overlap may delay the diagnosis and treatment of PPCM.

The etiology and pathophysiological mechanisms underlying the coexistence of PPCM and severe preeclampsia remain poorly understood. Patients with severe myocardial dysfunction and refractory hemodynamic instability are at particularly high risk of adverse outcomes. In such cases, venoarterial extracorporeal membrane oxygenation (VA-ECMO) can provide temporary circulatory support as a bridge to myocardial recovery. However, evidence supporting its use in PPCM complicated by severe preeclampsia is limited. Crucially, while short-term survival using VA-ECMO has been documented, detailed long-term cardiac outcomes remain scarce in the literature. Here, we report a critically ill primigravida with PPCM and severe preeclampsia who developed persistent hemodynamic instability after emergency cesarean delivery and was successfully treated with VA-ECMO. Distinct from previous reports, this case provides a comprehensive 5-year follow-up, documenting the complete clinical trajectory from an antenatal conduction abnormality with preserved systolic function to profound cardiac dysfunction, subsequent myocardial recovery, and sustained normal LVEF. This case underscores the importance of timely echocardiographic evaluation, multidisciplinary management, early consideration of mechanical circulatory support, and long-term cardiac surveillance. The patient provided written informed consent for the anonymous publication of her clinical information.

## Case presentation

**Figure 1:**
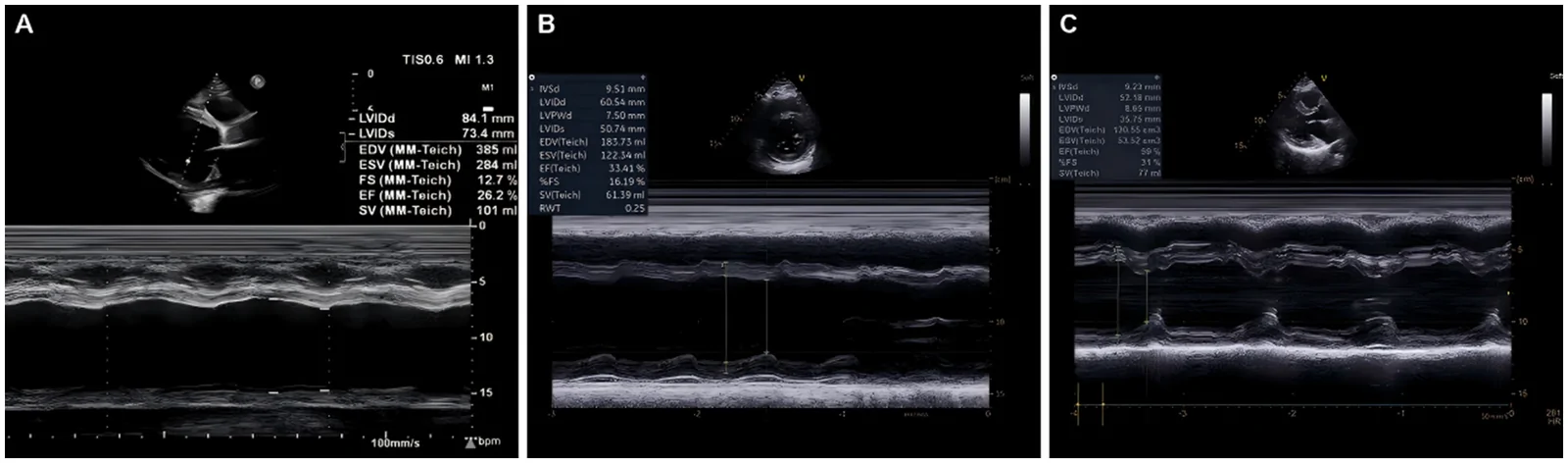
Serial transthoracic echocardiographic assessment of left ventricular systolic function. **(A)** At admission at 37 + 6 weeks of gestation, bedside echocardiography demonstrated severe left ventricular systolic dysfunction, with a LVEF of 26%. **(B)** On day 4 of VA-ECMO support, LVEF had increased to 33%, accompanied by hemodynamic stabilization and subsequent successful decannulation. **(C)** At the 5-year follow-up, LVEF was 59%, indicating sustained recovery of left ventricular systolic.

A 28-year-old primigravida (G1P0) was admitted at 37 + 6 weeks of gestation with a 2-day history of progressive orthopnea, dyspnea, and cough. At 15 + 3 weeks of gestation, electrocardiography had demonstrated sinus tachycardia and complete left bundle branch block. Echocardiography, however, showed preserved left ventricular systolic function, with a LVEF of 61%. She was asymptomatic at that time, and no specific intervention was recommended after cardiology consultation. Subsequent antenatal assessments were unremarkable, with normal glucose tolerance and blood pressure and no proteinuria or edema. At 35 + 0 weeks of gestation, her blood pressure was 134/75 mmHg. Her medical and family histories were otherwise unremarkable. On admission, she was orthopneic and unable to tolerate the supine position. Her temperature was 37.1 °C, heart rate 157 beats/min, respiratory rate 20 breaths/min, and blood pressure 159/120 mmHg. Bilateral pulmonary crackles and enlarged cardiac borders were noted. The fetus was in cephalic presentation, with a regular fetal heart rate of 145 beats/min, and the membranes were intact. Laboratory investigations showed an N-terminal pro-B-type natriuretic peptide (NT-proBNP) concentration of 5,660 pg/ml, a blood lactate concentration of 2.6 mmol/L, and proteinuria. Creatine kinase-MB, myoglobin, lactate dehydrogenase, creatine kinase, fibrinogen, and D-dimer levels were also elevated, whereas the prothrombin time and activated partial thromboplastin time were within their respective reference ranges. Bedside echocardiography revealed severe left ventricular systolic dysfunction, with an LVEF of 26% (Figure 1A), and thoracic ultrasonography demonstrated small bilateral pleural effusions. The initial diagnoses were preeclampsia with severe features and acute decompensated heart failure. Given the timing of presentation, the marked reduction in LVEF despite previously preserved systolic function, and the absence of an identifiable alternative cause, PPCM was considered the most likely etiology of heart failure.

Initial management included oral labetalol (100 mg every 8 h), intravenous magnesium sulfate for seizure prophylaxis, and intravenous furosemide (20 mg). An emergency cesarean delivery was performed, and a male infant weighing 2,880 g was delivered, with Apgar scores of 8 and 10 at 1 and 5 min, respectively. Postoperatively, the patient remained hemodynamically unstable and was admitted to the intensive care unit, where mechanical ventilation was initiated. She received intravenous furosemide (20 mg), deslanoside (0.4 mg), and levosimendan (12.5 mg by intravenous infusion). Additionally, norepinephrine (30 μg/min) were administered via continuous intravenous infusion. Despite ventilatory support and pharmacotherapy, hemodynamic instability persisted, with a central venous pressure (CVP) of 3–4 mmHg, serum lactate of 3.2 mmol/L, and markedly elevated NT-proBNP at 5,920 pg/ml. Blood pressure fluctuated between 80 and 125/60–100 mmHg, and heart rate ranged from 150–155 beats/min. Multidisciplinary evaluation supported the diagnosis of PPCM complicating preeclampsia with severe features, manifesting as cardiogenic shock consistent with INTERMACS Profile 1. After informed consent was obtained from the family, VA-ECMO was initiated as rescue circulatory support. Additional management included antimicrobial prophylaxis, transfusion of packed red blood cells and platelets, and prophylactic anticoagulation with nadroparin calcium (3,075 IU subcutaneously daily). By day 4 of VA-ECMO support, with norepinephrine and epinephrine successfully weaned, the patient was hemodynamically stable, with a blood pressure of 118/74 mmHg and a heart rate of 79 beats/min. Her CVP was 10 mmHg, blood lactate concentration had normalized to 1.1 mmol/L, NT-proBNP had decreased to 2,230 pg/mL, and LVEF had increased to 33% (Figure 1B). VA-ECMO flow was gradually reduced, and she was successfully decannulated later that day.

**Figure 2:**
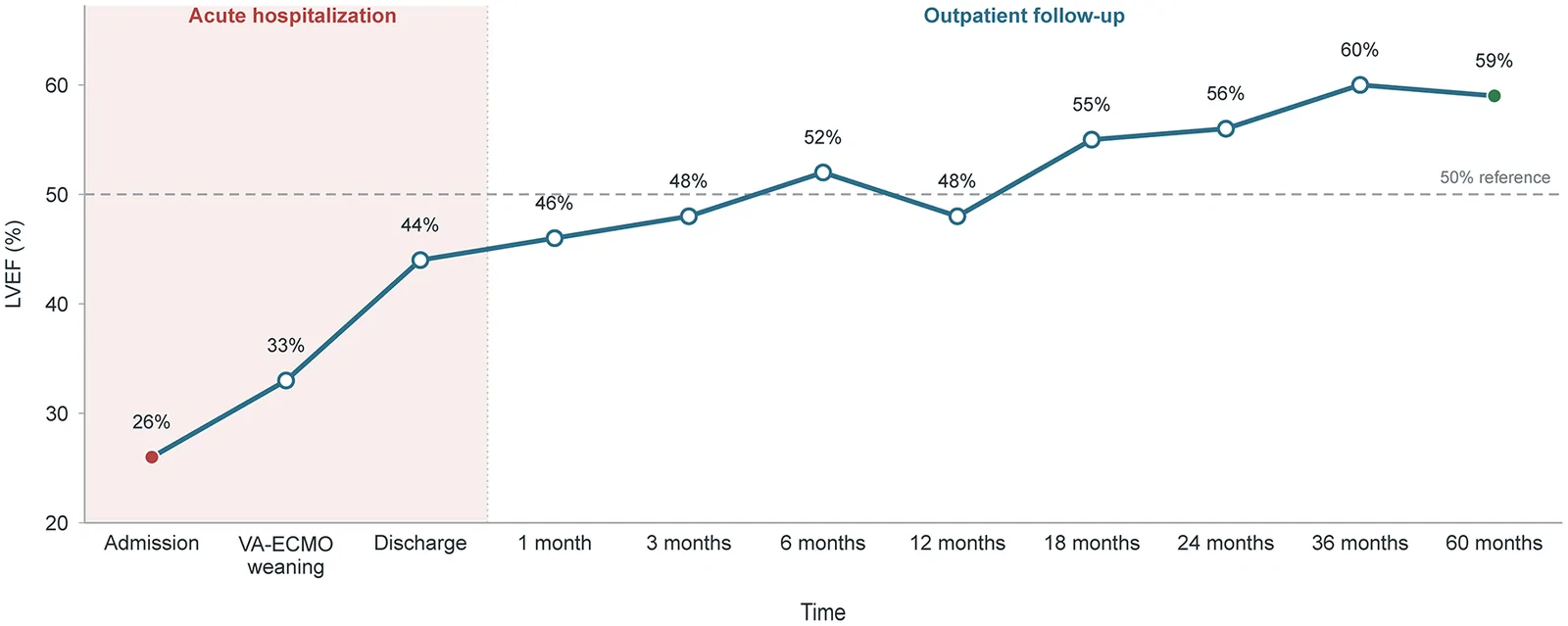
Longitudinal recovery of left ventricular systolic function after peripartum cardiomyopathy requiring VA-ECMO. LVEF was severely reduced at admission (26%), increased to 33% at VA-ECMO weaning and to 44% at discharge, and continued to improve during outpatient follow-up. Despite a transient decrease at 1 year (48%), LVEF subsequently recovered and remained near normal, reaching 59% at 5 years. The dashed horizontal line denotes an LVEF of 50%.

By postoperative day 9, the patient remained hemodynamically stable, with a blood pressure of 121/78 mmHg and a heart rate of 80 beats/min. NT-proBNP had decreased further to 977 pg/ml, and LVEF had increased to 46%. Respiratory support was reduced to low-flow oxygen administered through a nasal cannula, and she was transferred to the cardiology ward. On postoperative day 21, her blood pressure was 114/64 mmHg and her heart rate was 70 beats/min. NT-proBNP had decreased to 727 pg/ml, the bilateral pleural effusions had partially resolved, and LVEF was 44%. She was able to lie flat. Symptoms such as fatigue, shortness of breath, and chest tightness emerged following brisk walking but improved after rest, corresponding to New York Heart Association (NYHA) functional class II. She was discharged after substantial clinical improvement and subsequently underwent regular outpatient follow-up with serial echocardiography and NT-proBNP measurements. At discharge, she was prescribed a regimen comprising oral spironolactone 20 mg once daily, oral furosemide 20 mg once daily, oral trimetazidine 20 mg three times daily, oral digoxin 0.25 mg once daily, oral metoprolol 25 mg twice daily, and oral warfarin 3 mg once daily. She was advised to avoid breastfeeding and to use barrier contraception (condoms). Multidisciplinary preconception counseling involving both obstetric and cardiology teams was recommended before any future pregnancy. NT-proBNP normalized within 6 months after delivery, with no limitations in physical activity (NYHA class I). LVEF increased to >50% by 9 months, at which point physical activity remained unrestricted (NYHA class I) and warfarin was discontinued. 2 years postpartum, LVEF was 56%, allowing for the cessation of all anti-heart failure medications. At the 5-year follow-up, her NT-proBNP concentration was 20.9 pg/ml and her LVEF was 59% (Figure 1C), with NYHA class I status, indicating sustained biochemical and myocardial recovery. As of the latest follow-up, the patient has no immediate plans for conception. For clarity, the longitudinal recovery of left ventricular ejection fraction is illustrated in Figure 2, while the major clinical milestones are summarized chronologically in Table 1.

**Table 1:** Timeline of clinical course.

Time point	Clinical course
15 + 3 weeks' gestation	Sinus tachycardia and complete left bundle branch block were detected; the patient was asymptomatic, with preserved LVEF (**61%**).
15–35 weeks' gestation	Antenatal assessments remained unremarkable, without hypertension, proteinuria, or edema.
37 + 6 weeks' gestation	Admitted with a 2-day history of progressive orthopnea, dyspnea, and cough; BP was 159/120 mmHg and HR was 157 beats/min.
At admission	Proteinuria, NT-proBNP **5,660 pg/ml**, lactate **2**.6 mmol/L, bilateral pleural effusions, and severe LV dysfunction (LVEF **26%**) were identified. PPCM complicated by preeclampsia with severe features was suspected.
Emergency delivery	Labetalol, magnesium sulfate, and furosemide were administered, followed by emergency cesarean delivery of a healthy male infant.
Immediate postoperative period	Persistent hemodynamic instability required mechanical ventilation and intensive pharmacotherapy; VA-ECMO was initiated as rescue circulatory support.
VA-ECMO day 4	Hemodynamics stabilized, lactate normalized, NT-proBNP decreased to 2,230 pg/ml, and LVEF improved to 33%; VA-ECMO was successfully discontinued.
Postoperative day 9	NT-proBNP decreased to 977 pg/ml and LVEF increased to 46%; the patient was transferred to the cardiology ward.
Postoperative day 21	NT-proBNP decreased to 727 pg/ml, LVEF was 44%, and pleural effusions partially resolved; the patient was discharged.
Long-term follow-up	NT-proBNP normalized by 6 months, LVEF exceeded 50% by 9 months, and at 5 years NT-proBNP was 20.9 pg**/ml** with an LVEF of 59%.

## Discussion

PPCM is a rare, idiopathic form of cardiomyopathy that typically manifests as dilated cardiomyopathy with predominant systolic dysfunction (4). Its incidence varies substantially across geographic regions and ethnic groups, affecting an estimated 1 in 2,000 women worldwide (5, 6). Because its clinical manifestations can resemble normal physiological changes during pregnancy or other obstetric complications, PPCM is frequently misdiagnosed or overlooked (2, 7). Established risk factors include multiparity, a family history of cardiomyopathy, African ancestry, smoking, alcohol abuse, diabetes mellitus, hypertensive disorders of pregnancy, malnutrition, and advanced maternal age (8, 9). The novelty of this case lies in the successful management of severe PPCM complicated by severe preeclampsia and refractory acute heart failure, with a baseline LVEF of only 26%. A combination of emergency cesarean delivery, pharmacological therapy, and VA-ECMO resulted in substantial long-term cardiac recovery. At the 5-year follow-up, the patient's LVEF had improved to 59%, and NT-proBNP levels had normalized. This case highlights the diagnostic challenges posed by the coexistence of PPCM and severe preeclampsia, demonstrates the potential value of VA-ECMO in refractory cardiogenic shock, and emphasizes the importance of long-term follow-up in patients with PPCM.

Accurate diagnosis of PPCM in the setting of severe preeclampsia was one of the principal challenges in this case. Both conditions may present during the third trimester or postpartum period with overlapping manifestations, including dyspnea, pulmonary edema, and cardiac dysfunction. Consequently, PPCM may be mistaken for preeclampsia-associated heart failure. A meta-analysis by Bello et al. found that approximately 22% of PPCM cases were complicated by preeclampsia, more than four times the estimated global prevalence of preeclampsia (5%) (10). Similarly, the ESC EORP PPCM Registry reported preeclampsia in 25% of patients with PPCM, while hypertensive disorders, including isolated hypertension and preeclampsia, affected up to 40% of the cohort (11).

Recent studies have identified convergent pathophysiological mechanisms underlying PPCM and preeclampsia, including increased expression of placental antiangiogenic factors such as soluble fms-like tyrosine kinase 1 (sFlt-1), systemic endothelial dysfunction, oxidative stress, and microvascular impairment (12–14). Preeclampsia is frequently accompanied by symptoms including dyspnea, headache, epigastric pain, nausea, vomiting, and visual disturbances. Preeclampsia-associated cardiac dysfunction generally improves after delivery and effective blood pressure control, with LVEF typically preserved. In contrast, overt peripartum heart failure with an LVEF below 45% strongly suggests PPCM (1, 15). According to the 2025 ESC Guidelines, prior diagnosis of PPCM is associated with a substantial increase in the risk of adverse maternal outcomes upon subsequent pregnancy. The guidelines specifically highlight that preconception moderate-to-severe left ventricular systolic dysfunction (LVEF < 40%) significantly elevates the risk of further deterioration. Complementing these recommendations, findings from a PPCM registry analysis revealed that baseline LVEF, left ventricular end-diastolic diameter (LVEDD), Human Development Index (HDI), duration of symptoms, QRS duration, and a history of preeclampsia are independently associated with adverse prognosis (5, 15). Although the patient met the diagnostic criteria for severe preeclampsia at admission, with a blood pressure of 159/120 mmHg and proteinuria, her markedly reduced LVEF of 26% could not be explained solely by hypertensive cardiac dysfunction. Echocardiography was therefore pivotal in identifying underlying PPCM rather than attributing the heart failure exclusively to preeclampsia. The subsequent recovery of cardiac function during postpartum follow-up further supported the diagnosis, consistent with the characteristic recovery pattern of PPCM (16). Collectively, these findings indicate that PPCM was the primary cause of acute heart failure, whereas severe preeclampsia likely contributed to its onset and progression. Timely recognition is a major determinant of prognosis. Previous studies suggest that early diagnosis and appropriate management result in partial or complete recovery of cardiac function in more than half of affected women (17, 18). This case therefore underscores the essential role of echocardiography in the diagnosis of PPCM (5, 7).

From a therapeutic perspective, this case underscores the critical importance of multidisciplinary team (MDT) collaboration and mechanical circulatory support (MCS). The management of PPCM primarily involves guideline-directed medical therapy for heart failure, adapted to the peripartum period (5). An analysis of the Extracorporeal Life Support Organization registry reported an overall survival rate of approximately 64% and a successful weaning rate of 72% among patients with PPCM receiving VA-ECMO (19). In the present case, the patient had an initial LVEF of only 26%, and myocardial dysfunction persisted despite delivery and pharmacological treatment. The MDT therefore promptly initiated VA-ECMO as a bridge to myocardial recovery. By the fourth day of ECMO support, the N-terminal pro-B-type natriuretic peptide level had decreased substantially, and the LVEF had improved from 26 to 33%, enabling successful decannulation. These findings suggest that MCS not only maintains end-organ perfusion but also provides the injured myocardium with sufficient time to recover. By temporarily supporting cardiorespiratory function, VA-ECMO may facilitate myocardial recovery and serve as a critical rescue therapy for patients with severe PPCM (20).

The prognosis of PPCM is generally more favorable than that of other forms of heart failure with reduced ejection fraction, with recovery of cardiac function reported in 23–78% of patients (21, 22). Current guidelines and expert consensus define normalization of cardiac function as a LVEF of ≥50%. Recovery typically occurs within 6 months but may take up to 2 years in some patients (21). A large-scale analysis of 4,875 patients with PPCM found that 44.1% achieved LVEF recovery (≥50%) by 6 months, increasing to 58.7% by 12 months. These findings indicate that incomplete or delayed recovery remains common (23). In the present case, the patient's LVEF was 44% at discharge and subsequently increased to 59% over nearly 5 years of follow-up. Therefore, long-term follow-up and regular monitoring of cardiac function remain essential for patients with PPCM (24).

This report has several limitations. First, findings from a single case cannot be generalized to the broader population of patients with peripartum cardiomyopathy. Second, coronary angiography, cardiac magnetic resonance imaging, and genetic testing were not performed; therefore, occult cardiomyopathy and other rare etiologies could not be definitively excluded. Third, high-quality echocardiographic images could not be obtained because of technical constraints. Nevertheless, the availability of reliable long-term follow-up data provides valuable insight into the patient's cardiac recovery trajectory and partially mitigates these limitations.

## Conclusions

This case highlights the diagnostic challenge of peripartum cardiomyopathy complicated by severe preeclampsia. Timely echocardiography, multidisciplinary management, and early VA-ECMO may provide an effective bridge to myocardial recovery in patients with refractory hemodynamic instability. Sustained cardiac recovery over 5 years also underscores the importance of long-term follow-up.

## Data Availability

The original contributions presented in the study are included in the article/supplementary material, further inquiries can be directed to the corresponding author.
